# Differential Functional Changes of Nav1.2 Channel Causing *SCN2A*-Related Epilepsy and Status Epilepticus During Slow Sleep

**DOI:** 10.3389/fneur.2021.653517

**Published:** 2021-05-19

**Authors:** Pu Miao, Siyang Tang, Jia Ye, Jihong Tang, Jianda Wang, Chaoguang Zheng, Yuezhou Li, Jianhua Feng

**Affiliations:** ^1^Pediatric Department, Second Affiliated Hospital, Zhejiang University School of Medicine, Hangzhou, China; ^2^National Health Center and Chinese Academy of Medical Sciences Key Laboratory of Medical Neurobiology, National Clinical Research Center for Child Health, Children's Hospital, Zhejiang University School of Medicine, Hangzhou, China; ^3^Department of Neurology, Children's Hospital of Soochow University, Suzhou, China

**Keywords:** SCN2A, developmental epileptic encephalopathy, gain-of-function, treatment, status epilepticus during sleep

## Abstract

**Background:** Nav1.2 encoded by the *SCN2A* gene is a brain-expressed voltage-gated sodium channel known to be associated with neurodevelopment disorders ranging from benign familial neonatal infantile seizures (BFIS) to developmental and epileptic encephalopathy (DEE) and autism spectrum disorder. Interestingly, status epilepticus during slow sleep (ESES), which aggravates cognitive impairment, has been found in *SCN2A*-related epilepsy. However, the functional features and the relationship between *SCN2A* and ESES have not been researched.

**Method:** We herein investigated the functional consequences of an unpublished *de novo* V911A and the other two published variants in patients with *SCN2A*-related disorder and ESES by whole-cell patch-clamp studies in transfected HEK293T cells.

**Results:** The unpublished V911A and published K1933M variants detected in patients with DEE exhibited a profound gain-of-functional (GOF) change. Another published BFIS variant S863F significantly reduced current density as a loss-of-functional (LOF) change. The refractory epilepsy in the patient with V911A was controlled by using the precise treatment of oxcarbazepine (OXC) since the age of 3 months. ESES was found at 18 months during the seizure-free period. We finally chose an aggressive treatment for eliminating ESES by using methylprednisolone combined with levetiracetam and nitrazepam instead of the precise treatment of OXC.

**Conclusion:** Both GOF and LOF variants in the *SCN2A* gene can lead to ESES among the phenotypes of DEE and BFIS. We should monitor the electroencephalogram regularly in the patients with *SCN2A*-related epilepsy even during their seizure-free period.

## Introduction

The *SCN2A* gene can cause a wide array of phenotypes ranging from benign familial neonatal infantile seizures (BFIS) and developmental and epileptic encephalopathy (DEE) to neurodevelopmental disorders such as autism spectrum disorder (ASD) and intellectual disorder (ID) (1). *In vitro* functional studies in HEK293 cells revealed that pathogenic variants in the *SCN2A* gene could lead to both loss-of-functional (LOF) and gain-of-functional (GOF) changes in the Nav1.2 channel. *SCN2A*-related epilepsy (DEE and BFIS) is associated with different effects on channel function (2), and the mechanism behind this paradox is still unknown. Patients with seizure onset before the age of 3 months are often found with GOF missense variants, and they respond well to sodium channel blockers (SCBs). The specific development-dependent expression patterns of the Nav1.2 channel in myelinated and unmyelinated nerve fibers may contribute to the different ages at seizure onset (3 months) and clinical symptoms (3).

Interestingly, the *SCN2A* gene has also the second most pathogenic variants associated with electrical status epilepticus during slow-wave sleep (ESES) (4). ESES has a special epileptic discharge pattern that is clinically linked to different epileptic syndromes, such as the Landau–Kleffner syndrome and epileptic encephalopathies with continuous spikes and waves during slow sleep (ECSWS) (5–7). The underlying etiology is complex, including brain malformations, immune disorders, and genetic factors. Genetic factors play an important role in ESES. Multiple genes such as *GRIN2A, SCN2A, KCNB1, CNKSR2, KCNQ2, KCNA2, SLC6A1*, and *WAC* (7–14), especially genes of channelopathy, were reported (4). Case reports of *SCN2A, KCNB1, KCNQ2*, and *KCNA2* with ESES were related to infantile epileptic encephalopathies, which underlines that ESES is not a rare electroencephalogram (EEG) pattern among DEE patients.

Seizures related to *SCN2A* variants are often intractable with the use of multiple antiepileptic drugs (AEDs). However, infants with seizure onset earlier than 3 months of age could be treated precisely with SCBs (15). The paradox is that oxcarbazepine (OXC) and carbamazepine (CBZ) should be avoided when patients are found with ESES, as they may worsen ESES (16). This issue has not been discussed so far even though hundreds of pathogenic variants have been discovered. In this study, we analyzed data from a patient with an unpublished *de novo* heterozygous variant of V911A in *SCN2A* gene and ESES pattern of EEG. We studied the functional changes using *in vitro* experiments. Meanwhile, to further understand the relationship between *SCN2A* variants and ESES, we then verified the functional changes of the other reported variants with ESES. Our findings outlined the phenotype–funotype of patients with ESES and the pathogenic variants of the *SCN2A* gene.

## Methods and Materials

### Ethical Compliance

All procedures were approved by the Ethics Committee of the Second Affiliated Hospital of School of Medicine, Zhejiang University, China (Ethical code:2018-080). Written informed consent was obtained from the patient's parents for the publication of any potentially identifiable data included in this article.

### Plasmid Constructs

The cDNA of human Nav1.2α was purchased from Addgene, and human Nav β1 and β2 subunits were generously provided by Dr. S. C. Cannon and Dr. S. G. Waxman. Wild-type (WT) Nav1.2 was subcloned into a pCMV vector. Variants were introduced into pCMV-Nav1.2 using a ClonExpress II One Step Cloning Kit (vazyme).The Nav β1 and β2 subunits were subcloned into pIRES2-EGFP and pIRES2-mCherry vectors, respectively. The ORF of all plasmids were confirmed by sequencing full length before transfection.

### Cell Culture and Transfection

HEK293 cells were obtained from American Type Culture Collection (ATCC) and maintained at 37°C with 5% CO_2_ in Dulbecco's modified Eagle's medium supplemented with 10% fetal bovine serum (Gibco). Expression of hNav1.2 and the accessory β1 and β2 subunits was achieved by using transient transfection with Lipofectamine 2000 (Invitrogen). Electrophysiological recordings of both green and red fluorescent cells were made 24 h after transfection.

### Electrophysiology and Data Analysis

Whole-cell voltage-clamp experiments were used to examine the voltage-gated Na^+^ currents. All voltage-clamp experiments were performed at room temperature. The data were collected using an Axon multiclamp 700B, Digidata1440A (Axon Instruments). Patch pipettes were pulled and fire polished to reach a pipette resistance of 1.1–2.0 MΩ. The solution in the pipette contained 10 mM NaF, 110 mM CsF, 20 mM CsCl, 2 mM EGTA, and 10 mM HEPES with a pH adjusted to 7.35 with CsOH and an osmolarity adjusted to 310 mOsmol/kg with sucrose. The bath solution contained 145 mM NaCl, 4 mM KCl, 1.8 mM CaCl2, 1 mM MgCl2, 10 mM d-(+) glucose, and 10 mM HEPES with a pH adjusted to 7.35 with NaOH and an osmolarity adjusted to 310 mOsmol/kg with sucrose. To minimize voltage errors, we focused on data from cells expressing maximal peak Na^+^ current amplitudes between 1 and 8 nA. We used low-resistance pipettes and 90–95% series resistance compensation. The average series resistance in these cells was 1.8 ± 0.3 MΩ, and the estimated maximum voltage error of the recordings was 1.6 ± 0.4 mV. Cells were held at −120 mV, and sodium currents were evoked by a series of depolarizing pulses (100 ms) to potentials ranging from −80 to +90 mV in steps of 5 mV. Current densities were obtained by dividing the peak currents by the capacitance. The voltage dependence of activation was obtained by plotting the normalized conductance against test potentials with the equation G/Gmax = 1/[1 + exp(*V*_0.5_ – *V*)/*k*], where the Gmax is the maximum conductance, *V*_0.5_ is the half-maximal activation potential, and *k* is the slope factor. the curve is fitted to a Boltzmann function. The voltage dependence of fast inactivation was assessed by applying a double-pulse protocol: 500 ms pre-pulses were applied from −150 to 0 mV in steps of 10 mV and followed by a test pulse of −10 mV. The steady-state fast-inactivation curve was fitted using the Boltzmann equation (I/Imax = {1 + exp[(*V* – *V*_0.5_)/*k*]} – 1). Recovery from fast inactivation was assessed by a two-pulse recovery protocol with varying time intervals between a 500 ms inactivating pre-pulse and a test pulse of −10 mV. The time course of recovery from inactivation was fitted with a double-exponential function to generate τ1 and τ2. Data are presented as mean ± SEM. Statistical comparison between more than two groups was performed using one-way ANOVA followed by Bonferroni *post-hoc* test.

### Statistics Method

All values were expressed as means ± standard error of mean, and the one-way ANOVA by Bonferroni *post-hoc* test was used for statistical analysis.

## Results

### Clinical Report

**Figure 1:**
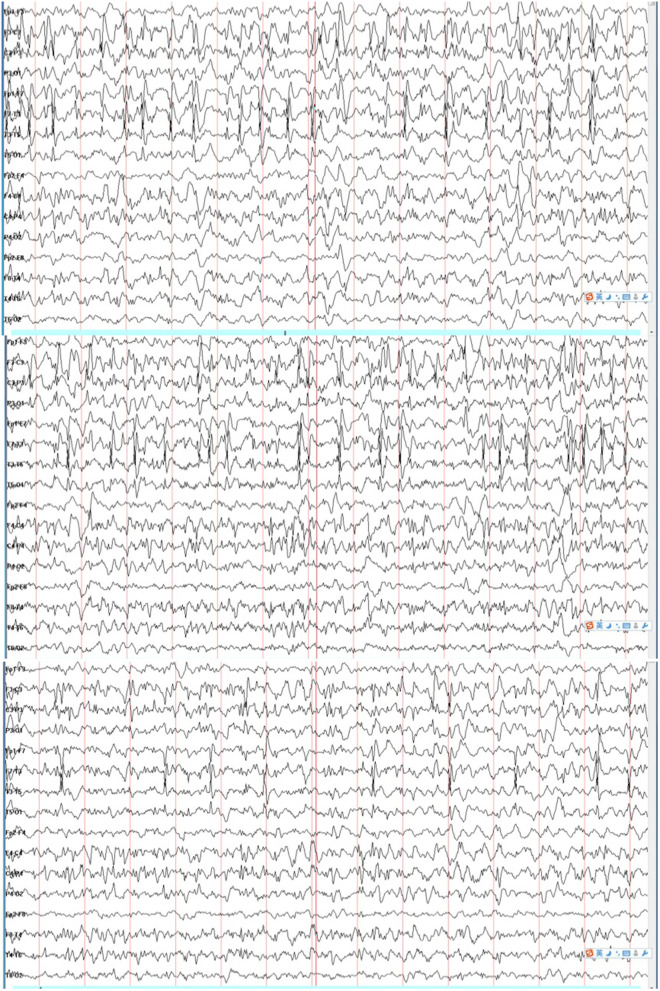
Electroencephalogram (EEG) showed electrical status epilepticus during sleep (ESES).

The patient was a 2-year-old boy and the first child of healthy and non-consanguineous parents. He was born at full term with a normal weight of 3,250 g. The delivery was eventful. Two days after birth, he began to display gradually increasing clonic seizures, from 2–3 times per day to 15–20 times per day. Blood test, cerebrospinal fluid biochemical tests, and cranial magnetic resonance imaging (MRI) findings were normal. Tandem mass spectrometry (MS/MS) of urinary and dry blood filter paper showed no significant abnormalities. The video electroencephalography (VEEG) showed a burst-suppression pattern 10 days after birth. The patient was first treated with phenobarbital (PB) and topiramate (TPM) and then with nitrazepam (NZP) and TPM because of intractable epilepsy. When the patient was about 3 months old, the result of a next-generation sequencing panel involving epilepsy-related genes showed a variant of c.2732T>C, p.V911A(NM_001040142). We validated this result with Sanger sequencing and confirmed the *de novo* type by testing the parents. It was predicted to be a pathogenic variant according to the American College of Medical Genetics and Genomics (ACMG) scoring, and a functional study confirmed that it was a GOF mutation which reinforced its potential pathogenicity. The patient was treated with OXC (25 mg/kg/day) and showed a remarkably good response since then. The VEEG re-examination showed generalized spike-slow waves instead of a burst-suppression pattern. Seizures have not reoccurred to this day. At the age of 1 year and 10 months, the Griffith Mental Development Scales (GMDS) indicated profound developmental delays for all six areas as follows: the patient could not sit well and say any words. During the VEEG re-examination, the patient displayed a spike wave index of 80% during non-rapid eye movement (NREM) sleep and of <10% during wakefulness at the age of 18 months (Figure 1).

The clinical examination made a conclusion of epileptic encephalopathy with continuous spikes and waves during slow sleep (CSWS). For patients with GOF mutations in *SCN2A*, SCBs should be considered as the first-line drug. However, for patients with CSWS, SCBs should be avoided. Ultimately, we considered withdrawing OXC and chose a more active treatment for continuous-wave square-wave (CSWS) with methylprednisolone (20 mg/kg/day for 3 days then 2 mg/kg/day orally for 4 days, three cycles above, then 1–2 mg/kg/day orally and gradually stop for 6 months), combined with levetiracetam (LEV) and NZP. The patient did not experience any seizures until the last follow-up, and the VEEG was normal after 4 months of our therapeutic schedule.

### The Functional Characterization of V911A and Reported K1933M and S863F

**Figure 2:**
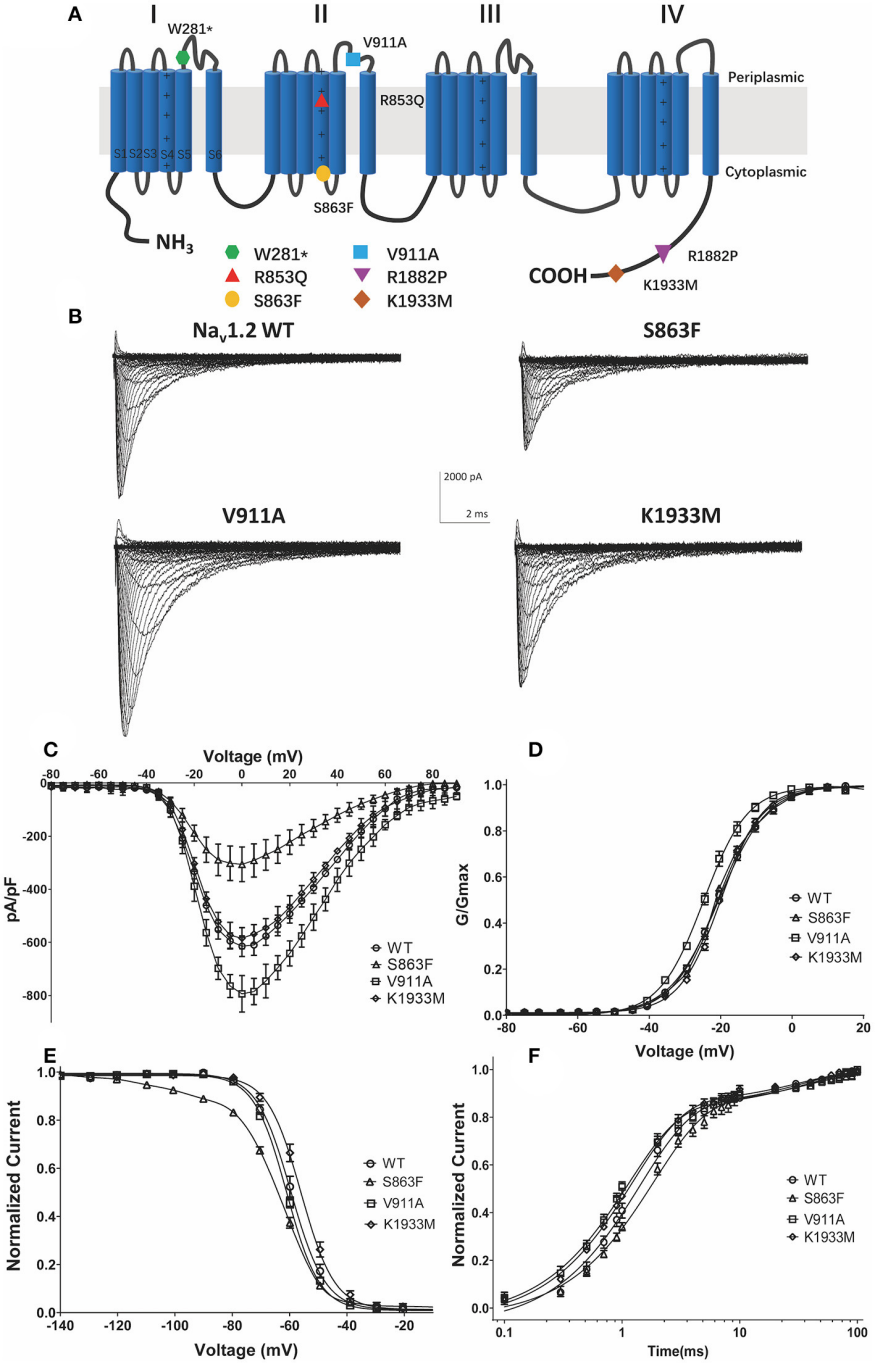
Functional studies reveal gain-of-function changes for p.(Val911Ala, V911A), p.(Lys1933Met, K1933M) variants, and loss-of-function changes for p.(Ser863Phe, S863F). **(A)** Transmembrane topology of the human Na_v_1.2 showing the location of the three functionally studied variants. **(B)** Representative whole-cell patch-clamp traces of voltage-dependent currents recorded from HEK293 cells transfected with either Nav1.2 wild-type or mutant channels **(C)**. Normalized I–V curves of peak sodium current density (in pA/pF) vs. voltage for WT and mutations. The current density of V911A mutant was significantly increased (*p < 0.05), and S863F mutant was significantly reduced (**p < 0.01) compared to WT **(D)**. Voltage dependence of channel activation. V911A revealed a significant hyperpolarizing shift of the activation curve compared to WT (**p < 0.01) **(E)**. Voltage dependence of fast inactivation. K1933M revealed a depolarizing shift, and S863F revealed a polarizing shift in the voltage dependence fast inactivation compared to WT (*p < 0.05) **(F)**. Time-dependent recovery from fast inactivation. V911A and K1933M revealed an accelerated recovery compared to WT; S863F slowed the recovery from fast inactivation (*p < 0.05). All fitting results are listed in Table 1.

The location of the variants in the Nav1.2 channel is depicted in Figure 2A. The S863F variant is located in S4 of domain II, V911A variant is located in the pore-forming loop of domain II, and the K1933M variant is in the C-terminal domain. We performed whole-cell patch-clamp recordings to determine the functional effects of the Nav1.2 channel variants. All mutated and WT channels were functional when transiently expressed in HEK293 cells. Figure 2B illustrates the representative whole-cell currents evoked by a series of depolarizing test potentials in cells transiently expressing WT, S863F, V911A, and K1933M Nav1.2. The corresponding current density–voltage relationship showed that the current density of V911A (−793.23 ± 68.67 pA/pF) was significantly increased compared to WT (−613.85 ± 38.72 pA/pF, Figure 2C). V911A channels also produced a hyperpolarizing shift in the voltage dependence of the activation curve. Compared to WT channels (−20.59 ± 0.83 mV), the half-maximal activation potential (*V*_0.5_) of V911A (−24.8 ± 0.91 mV) shifted by 4 mV in a hyperpolarized direction (Figure 2D). Moreover, the fast-inactivation recovery time constant τ1 obtained for V911A (1.04 ± 0.08 ms) was smaller than for WT channels (1.42 ± 0.11 ms) (Figure 2F and Table 1). The current density and the voltage dependence of activation of K1933M variants were not significantly changed compared to WT (Figures 2C,D). However, the fast-inactivation curve of K1933M channels exhibited a ~4 mV depolarizing shift of *V*_0.5_ (−56.10 ± 1.58 mV) as compared to WT (−59.67 ± 1.65 mV) (Figure 2E). Furthermore, K1933M also displayed a faster recovery from fast inactivation than WT, as indicated by the smaller fast time constant τ1 (WT, 1.42 ± 0.11 ms; K1933M, 1.07 ± 0.07 ms) (Figure 2F and Table 1). These results showed that V911A and K1933M variants induced GOF changes. On the other hand, S863F shifted the fast-inactivation curve to more negative potentials (−65.08 ± 0.95 mV), slowed the recovery from fast inactivation (τ1 = 1.85 ± 0.08 ms), and significantly reduced current density (−305.53 ± 65.15 pA/pF), indicated S863F was a LOF mutant.

**Table 1:** Functional characteristics of Nav1.2 channel.

	**Peak amplitude of Na^**+**^ current**	**Voltage dependence of activation**		**Voltage dependence of fast inactivation**		**Recovery from fast inactivation**	
	**pA/pF**	***V*_**0.5**_ (mV)**	***k* (mV)**	***V*_**0.5**_ (mV)**	***k* (mV)**	**τ1 (ms)**	**τ2 (ms)**
WT	−613.85 ± 38.72	−20.59 ± 0.83	6.84 ± 0.64	−59.67 ± 1.65	5.91 ± 0.84	1.42 ± 0.11	37.58 ± 8.62
S863F	−305.53 ± 65.15^**^	−20.80 ± 0.88^*^	6.60 ± 0.69	−65.0 ± 0.95^**^	9.07 ± 0.61^**^	1.85 ± 0.08^**^	41.30 ± 8.11
V911A	−793.23 ± 68.67^*^	−24.8 ± 0.91^**^	6.22 ± 0.55	−61.05 ± 0.74	5.73 ± 0.50	1.04 ± 0.08^*^	33.54 ± 6.02
I1571T	−572.07 ± 104.75	−27.57 ± 1.82^**^	6.70 ± 0.72	−59.60 ± 1.30	4.93 ± 0.47	0.61 ± 0.15^**^	25.02 ± 6.14^**^

*V_0.5_ is voltage of half-maximal (in-)activation; k is slope factor; τ is time constant. Data are presented as means ± SEM, n = 12–15*.

* *p < 0.05 and*

** *p < 0.01 vs. WT were determined by a Dunnett's post-hoc test after a one-way ANOVA*.

### Review of the Patient Phenotypes, Variants, and Functional Changes

There are six patients with six variants in the *SCN2A* gene that have been reported to have the ESES phenotype. Combined with our patient, six patients showed DEE, and one showed BFIS. Comprehensive phenotypes of the patients, bioinformatic annotations, and functional data of their variants are presented in Table 2.

**Table 2:** Patients' phenotype, molecular features, and functional results.

	**Patient 1**	**Patient 2**	**Patient 3**	**Patient 4**	**Patient 5**	**Patient 6**	**Patient 7**
Previous publication	Berecki et al. (17)	Wolff et al. (12)	Wolff et al. (12)	Wolff et al. (12)	Wolff et al. (12)	Wolff et al.	Ours
cDNA/protein change	R853Q	K1933M	R1882P	c.698-1G>T	W281*	S863F	V911A
Location in protein	DII S4	C-terminus	C-terminus	DI S4	DI S5–S6	DII S4	DII S5-6
Functional change	LOF	GOF	/	/	/	LOF	GOF
Phenotype	WS	DEE	DEE	DEE	DEE	BNIF	OS
Seizure onset	13 m	4 y	4 m	4 y 6 m	4 y 6 m	5 d	2 d
Seizure type	S,M	F, GTCS, Aab	T, C	F	F	GTCS SE F	C
MRI	N	N	N	N	N	N	N
EEG change	HA → MF spikes → ESES	MF → ESES	MF → ESES	F spikes → MF → ESES	MF → ESES	NA → MF → ESES	BS → MF → ESES
Effective AEDs	VGB, PB, CLB	ST, VPA, TPM, CLB, CS	LEV	TPM	ST	OXC-ST	OXC → M, NZP
Non-effective AEDs	PB, VPA, LTG, KD	LTG, LCM, CBZ	PB, VPA ST KD, LTG	/	/	/	/
Typical features	Movement disorder	ASD, ataxia	ASD, regression aggression	Mi-ID	M-ID	Attention deficit disorder	S-ID, ASD

***Phenotype:*** *WS, West syndrome; OS, Ohatahara syndrome*.

***Seizure type:*** *S, spasms; M, myoclonus; F, focal; GTCS, generalized tonic-clonic seizure; Aab, atypical absence; T, tonic; C, clonic; SE, status epilepticus*.

***EEG change:*** *MF, mutilfocal; HA, hypsarrhythmia; BS, burst suppression*.

***Typical features:*** *ASD, autism spectrum disorder; Mi-ID, mild intellectual disability; M-ID, moderate intellectual disability; S-ID, severe intellectual disability; MF, multifocal*.

***Drugs:*** *CBZ, carbamazepine; CLB, clobazam; KD, ketogenic diet; LCM, lacosamide; LTG, lamotrigine; LEV, levetiracetam; OXC, oxcarbazepine; PB, phenobarbital; PHT, phenytoin; ST, sulthiame; TPM, topiramate; VGB, vigabatrin; M, methylprednisolone; NZP, nitrazepam*.

## Discussion

ESES is a special type of EEG pattern with a prevalence of 0.5%, and structural or genetic abnormalities are the most common etiologies of ESES. *GRIN2A, SCN2A, KCNQ2, KCNB1*, and *KCNA2*, which encode significant channels in the brain, contributed to the etiology of ESES solely (4). However, the relationship between phenotype, genotype, and funotype among patients diagnosed as the *SCN2A*-related disorder and ESES has not been studied.

In addition to the six reported cases, we describe the seventh patient harboring a new *de novo* missense pathogenic variant in the *SCN2A* gene with ESES (12). We selected our patient's V911A variant and the published K1933M variant with DEE, as well as the S863F variant with BFIS for the further study of genotype–phenotype–funotype correlation. All these three variants from patients with *SCN2A*-related epilepsy and an ESES pattern here showed distinct functional changes. A recent study found GOF variants R230 and R227 in *KCNQ3* that showed multifocal status epilepticus during sleep (18). However, in our study, both DEE and BFIS phenotypes and both GOF and LOF variants in the *SCN2A* gene can combine with ESES.

Most functional studies revealed that variants detected in neonatal and early infantile epilepsies, including some DEE cases and almost all BFIE cases, lead to hyperexcitability of sodium channel activity (19). In our study, S863F was found in a patient with BFIS who had seizure onset at 5 days after birth, while the electrophysiological result of S863F exhibited LOF effects. S863F owns its particularity, and it is the only reported variant in *SCN2A* BFIS with ESES (12). R853Q, also in dominant II(S4), was detected in a patient with DEE and ESES, and a published study recognized it as another LOF variant (17). Protein truncating variant W281^*^ and splice variant C.698-1G>T, the other published variants in *SCN2A* with ESES, always lead to decreased neuronal excitability. These four LOF variants in *SCN2A* combine with the ESES phenotype. The detailed knowledge of basic phenotype mechanisms behind variant characteristics is obscure. Recent studies reinforced the understanding of epilepsy as a network disease (20, 21). Researchers have discovered that an impaired cortico-striatal excitatory transmission can trigger epilepsy among *SCN2A* haplodeficient mice. This shows that the networks are also crucial to the pathophysiology and genetic etiology of epilepsy (22). The overlap of the cortico-striatal-thalamus network between ESES (23) and *SCN2A* might explain the reason why LOF variants in the *SCN2A* gene showed ESES.

The major goal of ESES syndrome therapy should be to prevent or reduce associated cognitive and neurodevelopmental deficits. Age at onset, duration of ESES, etiology, and response to treatment are the possible predictors of outcomes (24, 25). The patient with the unpublished GOF V911A *de novo* variant displayed a typical phenotype–genotype–funotype, which at first led to no confusion about the use of SCBs as a valid treatment option. A patient's motor and cognitive abilities may worsen with the use of SCBs during an ESES pattern. Finding an effective treatment for these patients is an important and complex issue. OXC and CBZ should be avoided as they may worsen ESES (26). The contradiction was highlighted when the patients treated with OXC experienced aggravation of ESES symptoms at their final control. According to this, OXC therapy is not the best option for patients with ESES as it can cause persistent damage and worsen symptoms and foreseeable improvement of cognitive functions after remission. The treatment of ESES in *SCN2A* DEE patients may be more challenging for some patients as an early stop of the onset of ESES was a significant predictor of treatment success in all analyses (24, 27, 28). Steroids and surgery are the most effective treatments for ESES. We chose steroids initially, as our patient did not have a surgical indication. Two months after treatment with steroids, the patient's EEG did not show any improvements. We added levetiracetam and benzodiazepines actively, and 3 months after stopping OXC, the patient had remission of ESES. Therefore, for DEE patients with ESES, the treatment should be more active and intensive to shorten the duration of ESES.

## Conclusion

ESES is found in patients expressing the GOF or LOF variants of *SCN2A*. This indicates that we should monitor EEG, especially among seizure-free patients. For patients with GOF variants, we should reinforce a treatment combining methylprednisolone and benzodiazepines to decrease ESES symptoms.

## Data Availability Statement

The datasets presented in this study can be found in online repositories. The names of the repository/repositories and accession number(s) can be found here: NCBI BioProject, PRJNA716238.

## Ethics Statement

The studies involving human participants were reviewed and approved by the Second Affiliated Hospital of Zhejiang University. Written informed consent to participate in this study was provided by the participants' legal guardian/next of kin. Written informed consent was obtained from the individual(s), and minor(s)' legal guardian/next of kin, for the publication of any potentially identifiable images or data included in this article.

## Author Contributions

PM and JF designed the experiments. JT supplied and did the EEG test and CZ followed up the patient. ST and JY carried out experiments. YL and ST analyzed the experimental results. PM and JW analyzed the experimental data. PM, ST, and JY wrote the manuscript. All authors contributed to the article and approved the submitted version.

## Conflict of Interest

The authors declare that the research was conducted in the absence of any commercial or financial relationships that could be construed as a potential conflict of interest.
